# Use of Autologous Conditioned Serum (ACS) for Osteoarthritis Treatment in Horses: A Systematic Review of Clinical Data

**DOI:** 10.3390/vetsci10120707

**Published:** 2023-12-18

**Authors:** Simone Della Tommasa, Walter Brehm, Giacomo Farì, Andrea Bernetti, Annarita Imperante

**Affiliations:** 1 Department for Horse, University of Leipzig, 04103 Leipzig, Germany; brehm@vetmed.uni-leipzig.de; 2 Department of Biological and Enviromental Science and Technologies (Di.Ste.B.A.), University of Salento, 73100 Lecce, Italy; giacomo.fari@unisalento.it (G.F.); andrea.bernetti@uniroma1.it (A.B.); 3 Field Veterinary Surgeon, 70100 Bari, Italy; im_anna6@outlook.com

**Keywords:** autologous conditioned serum, osteoarthritis, horse, IRAP, joint

## Abstract

**Simple Summary:**

Regenerative medicine has emerged as a widely employed therapy for osteoarthritis (OA) and tendon lesions in both veterinary and human medicine. Despite numerous studies having been conducted, definitive outcomes remain elusive. This study seeks to comprehend the evolution of regenerative medicine from in vitro to in vivo studies, focusing on the effectiveness of Autologous Conditioned Serum (ACS) products in treating OA. The study only took into consideration clinical trials using ACS. All relevant articles published in the last two decades have been scrutinized. Despite a plethora of in vitro studies, the positive outcomes of autologous conditioned serum (ACS) products in in vivo settings have not been conclusively demonstrated. Notably, only six studies met the inclusion criteria. One study incorporated a control group, whereas the other five clinical trials were conducted without a control group. Consequently, there exists no definitive evidence supporting the efficacy of ACS therapy. This underscores the need for more controlled trials to draw a firm conclusion about the effectiveness of ACS as an OA treatment in horses, a conclusion that would have potential implications for humans. The current scarcity of controlled studies highlights the imperative for further research to enhance our understanding of ACS therapy’s therapeutic potential in addressing osteoarthritis.

**Abstract:**

The utilization of Autologous Conditioned Serum (ACS) for treating osteoarthritis (OA) in horses has seen a notable increase in recent years. In vitro studies have consistently identified ACS as a promising therapy for OA joints, contributing to its growing popularity in OA treatment. Despite this, there is a noticeable absence of systematic reviews focused solely on the clinical data of OA patients treated with ACS, excluding the in vitro perspective. This study aims to address this gap by systematically reviewing the latest literature, concentrating solely on clinical data in in vivo studies to evaluate the efficacy of ACS in OA lesions. All clinical studies involving ACS treatments for horses with OA were included in the assessment. Surprisingly, only six trials met the inclusion criteria for this systematic review. The results indicate that the majority of the considered articles support the use of ACS as a treatment for horses, albeit with a control group provided in only one study. However, the absence of a control group and the exclusion of histological evaluation diminish the validity of the majority of clinical research. While several studies suggest a beneficial effect of ACS on OA horses without significant adverse effects, this systematic review affirms that there is no definitive evidence for its effectiveness. Therefore, further investigation of the efficacy of ACS products as a treatment for OA is warranted, emphasizing the need for more controlled trials. Poorly designed and biased studies, lacking blinding or control and adopting inadequate outcome measures, may favor positive results and, thus, necessitate a more rigorous approach to validate the efficacy of ACS in OA treatment.

## 1. Introduction

Osteoarthritis (OA) poses a significant challenge in horses, particularly impacting athletic and juvenile cohorts, with profound implications for both prevalence and the economic landscape within the equine industry [1]. This complex issue stems from singular or repetitive trauma, which initiate a cascade of pathological processes that underlie the inevitable progression toward OA [1,2,3] The consequences are extensive, leading to prolonged absences from athletic pursuits and imposing a considerable burden on rehabilitation efforts.

Within the domain of OA treatment, the central objectives revolve around addressing the dual challenges of mitigating cartilage degradation and orchestrating the nuanced management of synovial and joint inflammation. Recent advancements have introduced novel biological therapies as treatment options for equine OA, among which Autologous Conditioned Serum (ACS) stands out [2,3,4]. The preparation of ACS involves incubating equine blood with borosilicate glass beads. The principal effects of ACS products include increased concentrations of interleukin-1 receptor antagonist (IL-1Ra, a competitive protein antagonist of the inflammatory cytokine IL-1β), and the anti-inflammatory cytokines IL-10 and IL-4 [4,5].

Various techniques, such as IRAP I™ (Dechra Veterinary Products/Orthokine) and IRAP II™ (Arthrex GmbH, München, Germany), are employed in veterinary medicine to obtain ACS products. The efficacy of ACS has been extensively explored through in vitro studies, in which its influence on growth factors and on anti-inflammatory and inflammatory cytokines has been examined [3,6,7]. Consequently, ACS has become a widely adopted option in OA treatment in veterinary medicine, particularly in horses. Despite this widespread use, the specific mechanism of action remains incompletely understood due to the diverse bioactive ingredients in different products and the actual therapeutic effects in OA joints [8,9,10].

Notably, only a limited number of studies have incorporated control groups (such as that of Frisbie et al. [2]). In this blind study focusing on induced OA of the carpus, horses treated with four ACS injections at weekly intervals exhibited reduced lameness scores, synovial thickness, and cartilage fibrillation compared to the control group receiving saline injections [2]. While clinical improvements are evident, in vitro studies have not consistently demonstrated chondroprotective effects, suggesting that the mitigation of inflammation primarily underlies the observed reductions in lameness and cartilage degradation [5]. Cumulatively, these results indicate that ACS predominantly functions as a mild anti-inflammatory agent in the joint [6,7].

Surprisingly, controlled studies employing ACS therapy remain scarce. Frisbie et al.’s [2] investigation, with histological assessments indicating a reduction in the rate of cartilage degradation, remains one of the few with a control group. Despite the dearth of controlled trials, ACS has become one of the most widely used incubating blood products for OA treatment based on its favorable outcomes observed in in vitro studies. Given this context, it is pertinent to compare the diverse outcomes published in studies using ACS therapy for OA treatment to gain insights into potential improvements in OA symptoms based on clinical experience. Hence, the primary objective of this study is to conduct a systematic review encompassing all clinically relevant studies, aiming to evaluate whether there is a significant improvement in OA symptoms after ACS treatment.

## 2. Materials and Methods

### 2.1. Selection of Studies for Systematic Review

All articles published between 2000 and 2022 were considered for inclusion. A comprehensive literature search on the use of Autologous Conditioned Serum (ACS) and IRAP in horses with osteoarthritis (OA) was conducted, encompassing studies published in English, Spanish, German, and Italian during the specified timeframe.

**Figure 1:**
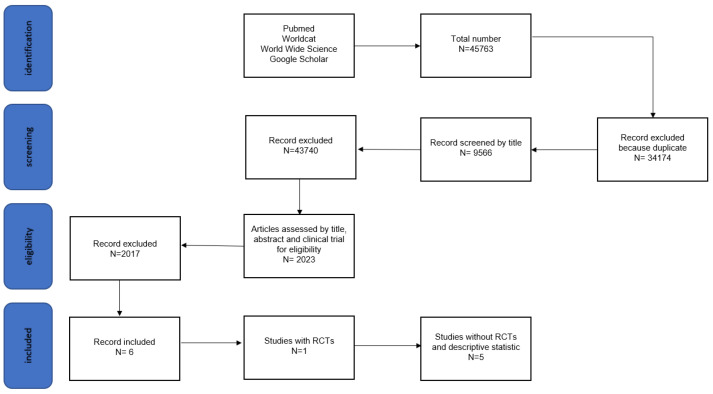
Flowchart depicting criteria for selection of studies.

The search criteria involved terms, such as “autologous conditioned serum”, “Osteoarthrosis”, “joint”, “horse”, “lameness”, “degenerative joint disease”, and “equine”. These terms were scrutinized in the full manuscript, abstract, title, and keywords of publications using web search engines indexing the full text or metadata of scholarly literature, including platforms, like PubMed, Worldcat, Wide Science, and Google Scholar. Duplicate articles were systematically excluded utilizing the EndNote program^®^ (Figure 1).

Exclusion criteria were applied to eliminate studies lacking horses, using biomaterials other than ACS, studying diseases other than OA, or in vitro studies. Clinical trials, regardless of their evidence level or design, were included due to their scarcity, with no distinction between studies with or without a control group.

To adhere to standard evidence-based medicine practices, the PICO model was used to guide the selection of studies for the review. The PICO model, summarized in Table 1, framed the study question: “In horses with OA problems, is the application of ACS products effective in reducing joint pain and improving prognosis?”.

### 2.2. Data Selection

The included studies included were selected based on all of the aforementioned characteristics. For each trial incorporated into the systematic review, the following data were extracted: year of publication, authors, journal of publication, type of intervention, treated joint, randomized controlled trials (RCTs) or not (No-RCTs), sample size, outcomes, and adverse effects.

Utilizing a pre-piloted data extraction form, two authors (SDT and AI) independently conducted the extraction of data. The inclusion criteria encompassed RCTs and No-RCTs utilizing Autologous Conditioned Serum (ACS) therapy for the treatment of osteoarthritis (OA) in adult horses. The studies involved horses affected by OA in various joints. Due to the limited number and heterogeneity of the studies, no restrictions were imposed on diagnostic methods or criteria, duration of the injury, number of injections, follow-up period, or evaluation of the outcome.

In horses with osteoarthritis (OA) problems, the study question (meticulously formulated using the PICO model and summarized in Table 1) seeks to determine the effectiveness of Autologous Conditioned Serum (ACS) products in reducing joint pain and improving prognosis. The comprehensive breakdown involves a clearly defined population (“Horses with OA problems”), a specific intervention (“Application of ACS products”), and the desired outcomes (“Reducing joint pain and improving prognosis”). Additionally, the inquiry incorporates the comparison aspect, exploring whether the effectiveness of ACS products can be established by contrasting them with a placebo or a standard treatment. This nuanced inclusion enhances the research question’s depth, acknowledging the importance of comparative analysis within the context of equine osteoarthritis. The precise application of the PICO model ensures a systematic and focused approach, aligning seamlessly with the fundamental principles of evidence-based medicine. The encapsulation of these elements within the research question not only provides clarity but also offers a structured and comprehensive inquiry into the potential benefits of ACS products, considering their efficacy in comparison to alternative treatments for equine osteoarthritis.

## 3. Results

Out of the initial pool of 45,763 studies, the exclusion process led to the removal of 34,174 duplicates. Subsequently, 9566 studies underwent screening based on their titles, and 2017 studies were further scrutinized based on clinical trial criteria. Following this, the inclusion criteria were applied to the remaining studies, leaving a final selection of six articles for closer examination (as detailed in Table 2). Among these final studies, one was classified as an RCT (Randomized Controlled Trial, study 1), while the remaining five were categorized as No-RTCs (Non-Randomized Controlled Trials, studies 2–6).

The selected clinical trials took into consideration the effects of ACS on OA horses. The joints treated during the studies were not indicated in all of them. The joints treated in studies 1, 2, 4, and 5 were 24 carpus, 7 fetlock, 26 coffin, 2 talocrural joints, and 1 femoro-tibial joint. Only studies 1 and 4 evaluated the outcome of the ACS therapy on the same joint. Studies 2, 3, 5, and 6 considered different joints in the study design.

All the studies used a different protocol for the injection of ACS. Study 1 injected ACS three times with an injection interval of 7 days. Study 2 had two schemes for two different groups: the first group received three injections at an interval of 7 days, and for the second group, the injection interval was every second day for three injections. Study 3 used 2–3 injections at an interval of 8–12 days (it was not specified which horse received 2 versus 3 injections, nor for which horses an 8- versus 12-day interval was used). Study 4 used 2–4 injections at intervals of 1 week (it was not specified when the different times of injections were used and in which horses). In study 5, two to three injections were performed at an interval of 7 days (no further details were given). Study 6 was conducted with four injections at intervals of 1 week.

Only two studies (studies 1 and 2) took into consideration the level of IL-1Ra concentration in the synovial fluid. In both groups, the IL-1Ra levels decreased. In study 1, the IL-1Ra decreased but they did not take into consideration the time in which the IL-1Ra decreased. In study 2, the IL-1Ra concentration increased in group 1 after 1 h injection, and 1 week after injection, values returned to the baseline. Also, in this study, group 2’s IL-1Ra levels increased 2 h after injection and decreased 48 h after. Synovial fluid IL-1Beta decreased 1–4 h after injection. Based on this outcome, the treatment protocol at 2-day intervals appears to be preferable for study 2.

Study 1 showed that ACS treatment reduced synovial membrane hyperplasia and cartilage fibrillation (or hemorrhage). This is the only study with a histological examination postmortem of the treated joints.

The follow-up interval is from 6 weeks to 5.8 years, and the results regarding the improvement in lameness are differently reported across the studies. In study 1, the lameness improved 70 days after injection, although no scale was used for the assessment. Similar results were reported in study 2, in which the lameness improved, but no scale was reported, nor was the time taken to improvement. In study 3, 199/262 horses were not lame after 6 weeks, 22 were not lame after 12 weeks, and 41 did not exhibit improvements in lameness. In study 4, 12 horses improved their lameness and 14 did not, but the time in which they improved the lameness was not indicated. In study 5, 6/12 horses improved their lameness after the first injection, and 10/12 horses improved after the second injection. In study 6, all horses recovered from lameness at 3 months post-injection. In total, of the 344 horses treated with ACS, 279 (81.1%) improved their lameness. This improvement in lameness was achieved by following different protocols and having different follow-up times. Lameness improvement was defined without taking into consideration a scale in all studies.

Side effects due to the ACS injections were observed in 5 horses (1.45%), starting from 2 h after injection and visible until 3 days after injection. The joint involved with side effects was always the coffin joint (5/5). Side effects included local pain and increased warmth of the injected joint and surrounding tissues. The authors assumed that side effects were probably caused by incorrect inoculation into the treated joint.

## 4. Discussion

Osteoarthritis (OA) poses a significant challenge in equine health, necessitating effective therapeutic interventions. Over the past 20 years, Autologous Conditioned Serum (ACS) has emerged as a promising treatment avenue. This novel approach involves extracting the horse’s own blood, processing it to concentrate anti-inflammatory and regenerative factors, and re-injecting it into the affected joints. While various in vitro and in vivo studies have explored the mechanisms of action of ACS and its role in mitigating inflammation during OA [2,3,4,7,8], there remains a critical need for comprehensive clinical trials to substantiate its efficacy and safety. Extensive research efforts have been dedicated to unraveling the intricacies of ACS in the context of OA. In vitro studies have provided valuable insights into the biochemical composition and potential mechanisms of action of ACS. These studies help elucidate how ACS may modulate inflammatory pathways and contribute to joint homeostasis. However, the transition from in vitro findings to in vivo and clinical applications has not been exhaustive. Clinical trials, particularly those involving ACS products, have been limited in number and scope. While existing studies endorse ACS as an effective treatment in equine practice, the lack of thorough exploration in clinical trials raises questions about the translational potential of in vitro discoveries.

This systematic review of the available literature aimed to shed light on ACS’s effects on clinical lameness evaluation. The evaluation process considered the heterogeneity of study designs, categorizing them based on their risk of bias. Surprisingly, the overall results did not reveal a clear correlation between bias risk and study conclusions. However, a glaring limitation was the absence of control groups in the majority of clinical studies, compromising the scientific rigor of the research. Each manuscript was evaluated, and the heterogeneity of the study designs was taken into account.

The inclusion of studies with a high risk of bias introduced heterogeneity into this review sample, affecting the overall reliability of the findings [16,17]. Moreover, the lack of blinding procedures and the predominant reliance on subjective assessments as primary outcomes further challenge the robustness of the available evidence. Low-quality studies were almost always associated with a positive ACS performance [18]. The majority of articles considered in this systematic review support the use of ACS as a treatment for horses, despite the absence of a control group in all but one study. Consequently, the lack of a control group and the exclusion of histological evaluation diminish the validity of the most clinical research [16,17].

A controversial topic emerging from the reviewed literature is the optimal protocol for ACS treatment. Different studies employed varying injection protocols, and only a limited number measured the concentration of the interleukin-1 receptor antagonist (IL-1Ra) after injections. The available evidence suggests that a protocol involving ACS treatments every second day may reduce synovial IL-1Ra concentration and improve lameness in horses. However, the limited number of studies in which IL-1Ra levels were measured calls into question whether this protocol is truly superior. This is especially true as protocols varied between studies, limiting comparisons of horses between studies with different protocols. Additionally, the joints need to be treated differently depending on the grade of inflammation. However, the optimal protocol remains elusive and likely depends on individual cases, as well as the degree of inflammation in the joints. 

Ambiguities regarding potential side effects linked to periarticular ACS application remain unresolved due to the limited number of studies and the absence of control groups. Side effects associated with ACS injections were limited to one study, and these were primarily linked to the treatment of the coffin joint. The authors suggested a correlation between side effects and the periarticular application of ACS, but they acknowledged the possibility of the distal interphalangeal joint being particularly sensitive to ACS injection due to its complexity. The absence of the control group and histologic examination leaves doubt as to whether the side effects are due to the use of ACS. The observed sensitivity to specific joint types raises questions about the generalizability of ACS treatment across various joint locations. Understanding the nuances of joint-specific responses to ACS could inform more targeted and effective treatment strategies.

Post-mortem examinations conducted in one study hinted at potential improvements in cartilage and synovial membrane macroscopic scores following ACS treatment. However, more studies exploring postmortem changes post-ACS treatment are necessary to render these findings more conclusive [2]. Histological examinations and postmortem assessments are crucial in providing a holistic understanding of the structural changes induced by ACS treatment [17,19,20,21]. Further research in this direction is necessary to ascertain the long-term effects and potential risks associated with ACS. 

The significance of the synovial membrane in the pathogenesis of OA has been highlighted in the literature. It is posited that the synovial membrane serves as the primary target of ACS products [22]. While ACS appears effective in reducing synovitis and inflammatory cytokine production, its direct impact on cartilage regeneration remains uncertain. In fact, according to previous studies, other tissues might be the main target for ACS actions in articular injuries because, for example, ACS had no impact on cartilage metabolism [17,19,20,21]. For instance, it has been established that the synovial membrane is a rich source of the inflammatory mediators and proteolytic enzymes that feed the chain of harmful intra-articular events that result in cartilage destruction [22]. As synovitis has been implicated in playing a significant role in OA, synovial tissues could be significant targets for the treatment of this condition [2,23,24,25,26]. Frisbie et al. [2] found a reduction in synovial membrane intimal hyperplasia in ACS-treated OA joints. Because the synovial membrane is involved in the initiation, progress, and persistence of OA (as well as its general pathogenesis) [26,27], and since the ACS products tend to reduce hyperplasia and the concentration of inflammatory cytokines into the synovial fluid, the main target of the ACS products could be the synovial membrane. The effects of the ACS products on the in vitro study also showed an improvement in cartilage regeneration [19]. Because the improvement in the cartilaginous surface could be directly correlated to the reduction in the inflammatory cytokines into the synovial fluid, the main target of the ACS products could be the synovial membrane [21,22,23,27,28].

The synovial membrane plays a crucial role in OA pathogenesis and is likely a key target for ACS products. This assumption suggests that ACS products may primarily alleviate synovitis and reduce inflammatory cytokine production, rather than directly promoting cartilage regeneration in joints with extensive cartilage damage [2,21,22]. In addition, the effects on articular cartilage are unlikely sufficient to support disease-modifying osteoarthritic drugs’ effects at this time [6,7].

Several limitations were identified in this systematic review, such as the absence of a control group, the lack of specification of different group characteristics (e.g., age, breed, and sex), and significant variations in joint type, numbers of injections, lameness evaluation scales, and follow-up durations across the evaluated trials. Most studies also lacked blinding evaluation, with subjective assessments (e.g., pain and lameness) being the primary outcomes. The absence of blinding procedures tends to exaggerate treatment effects, and the variability in operator knowledge of injected substances may influence interpretation. Non-blinded studies, with poor standardization evaluation of the outcome, are unreliable because the participants tend to interpret the results in a positive way. In addition, often, operators are aware of which substance they are injecting; in fact, placebo (usually sterile saline solution) and ACS have an obviously different appearance, which gives away their identity to those injecting them. A useful solution to this problem would be to have two different operators injecting the substances and examining the horses.

Investigations incorporating positive control groups could provide a benchmark for assessing the efficacy of ACS and offer insights into its potential advantages or limitations compared to conventional anti-inflammatory therapies.

## 5. Conclusions

The discourse surrounding Autologous Conditioned Serum (ACS) in the context of osteoarthritis (OA) treatment for horses has unraveled a multifaceted landscape, encompassing its mechanisms, clinical applications, and inherent limitations. Despite a wealth of in vitro studies delving into the biochemical intricacies and potential mechanisms of ACS, a significant void remains in bridging these laboratory findings to practical in vivo and clinical applications.

This study highlights the intricate landscape of ACS therapy for OA in horses. This systematic review, the first of its kind to exclusively consider clinical studies, provides a comprehensive overview of the current status of ACS applications in equine medicine. However, it also underscores the inherent challenges in the existing body of literature.

The heterogeneity observed among the studies introduces a significant risk of biases, complicating the interpretation of results and emphasizing the need for methodological standardization. The lack of control groups, blinding procedures, and reliance on subjective assessments in many studies point to the urgency of conducting well-designed clinical trials. Future research endeavors should prioritize blinded control studies with rigorous methodologies to elucidate the true effects of ACS products on lameness and other OA symptoms in horses. Notably, efforts must be increased to bridge the gap between in vitro studies exploring ACS’s biochemical intricacies and its practical in vivo and clinical applications. Standardization emerges as a key theme, urging researchers to establish clear and uniform protocols, reduce heterogeneity, and define standardized outcome measures, especially in lameness evaluations.

Despite the widespread use of ACS in equine medicine, this review highlights the ambiguity surrounding the main targets of ACS products. While the synovial membrane’s role is hypothesized, further investigations are crucial to delineate specific mechanisms and primary targets within the intricate joint environment of horses. This knowledge gap is pivotal for refining treatment protocols and maximizing the therapeutic potential of ACS in managing equine osteoarthritis.

This study also emphasizes the need for postmortem examinations and histological assessments to provide a holistic understanding of the structural changes induced by ACS treatment. Exploring the long-term effects and potential risks associated with ACS is essential for informed decision making in equine orthopedics.

In summary, this systematic review unravels the complexities and opportunities associated with ACS therapy in equine osteoarthritis. Moving forward, a concerted effort toward methodological rigor, standardization, and a deeper exploration of ACS mechanisms is imperative to enhance the evidence base. Efforts towards these ends will ultimately contribute to more effective and reliable treatment strategies for equine OA.

## Figures and Tables

**Table 1:** PICO question used to select the different study.

P	I	C	O
Population	Intervention	Control	Outcome
Horse with OA problem causing lameness	ACS Therapy	Placebo group in RCT	Improving of lameness

**Table 2:** Summary of all studies included in the systematic review.

	Title of the Study	Number of Horses	Methods	Injections Interval (days)	Follow-Up Period	Improvement of Lameness	Side Effects	Joint
**1**	Clinical, biochemical, and histologic effects of intra-articular administration of autologous conditioned serum in horses with experimentally induced osteoarthritis Frisbie et al., 2007 [2]	16	8 placebo (NaCl) 8 ACS	0–8–16–24	70 days	improvement of lameness; reduction of membrane hyperplasia; less cartilage fibrillation/haemorrhage; IL-1 ra decreased	no	carpus
**2**	Evaluation of two protocols using autologous conditioned serum for intra-articular therapy of equine OA—a pilot study Lasarzik et al., 2016 [11]	12	2 groups, 3 injections; first injection 14d after arthroscopy	(1) 0–8–16 (2) 0–2–4	42 days	Improvement of lameness; group 1 the IL-1ra concentration increased after 1h injection; 1 W after injection values returned to the baseline before the injection; group 2 IL-1ra increased 2h after injection and decreased 48h after. SF IL-1BEta decreased 1-4h after injection. The treatment protocol at 2-day intervals appears to be preferable.	no	3 metatarso/carpo-phalangeal 2 Tibio-tarsal 1 Femoro-tibial
**3**	Klinische erfahrung mit der Anwendung von ACS Weinberger 2008 [12]	262	2–3 injections	8–12	6–12 Weeks	199 improvement of lameness after 6 weeks; 22 no lameness after 12 weeks; 41 no improvement of the lameness	no	Not specified
**4**	Treatment of Osteoarthritis with ACS (IRAP^®^) on 26 horses—retrospective study Warner et al., 2016 [13]	26	2–4 injections	7	4,1-year average (2,4–5,8)	8 return to the previous or higher level; 4 same level; 14 no improvement	no	Distal interphalangeal joint
**5**	The success of IRAP treatment in DJD in Swedish racehorses Platvoet et al., 2011 [14]	12	2–3 injections	7		6 horses did not improve the lameness after the first injection. After the second injections 10 horses improved the lameness	no	8 carpus 4 metatarso/carpo-phalangeal
**6**	First result on the outcome of gold-induced, autologoous-conditioned serum in the treatment of different lameness-associated equine disease Schneider et al., 2013 [15]	16	4	7	3–6 months	all the horses were not lame at 3 months after injection	5 horses showed local pain after 2 h until 3 days after injection	Not specified

## Data Availability

No new data were created.
